# Morphology of the oldest fossil subfamily of Limoniidae (Diptera, Architipulinae) in the light of exceptionally preserved Mesozoic material

**DOI:** 10.1038/s41598-021-03350-4

**Published:** 2021-12-17

**Authors:** Katarzyna Kopeć, Agnieszka Soszyńska-Maj, Iwona Kania-Kłosok, Robert A. Coram, Wiesław Krzemiński

**Affiliations:** 1 Institute of Systematics and Evolution of Animals, Polish Academy of Sciences, 31-016 Kraków, Poland; 2 Department of Invertebrate Zoology and Hydrobiology, Faculty of Biology and Environmental Protection, University of Lodz, 90-237 Lodz, Poland; 3 Department of Environmental Biology, University of Rzeszów, Zelwerowicza 4, 35-601 Rzeszow, Poland; 4 School of Earth Sciences, University of Bristol, Bristol, BS8 1RJ UK

**Keywords:** Entomology, Palaeontology, Taxonomy

## Abstract

Based on known fossil evidence the extinct subfamily Architipulinae is considered to be the oldest evolutionary group of the Limoniidae, the largest family within the infraorder Tipulomorpha. The morphology of this subfamily, which includes 11 genera, has so far been based mainly on wing venation. New well-preserved representatives of the genus *Cretolimonia* Kalugina, 1986 were recovered from the Jurassic/Cretaceous boundary of Shevia and Daya, Transbaikalia, as well as from mid-Cretaceous amber from Kachin, Myanmar. This new material enriches our knowledge of the subfamily Architipulinae and of the genus *Cretolimonia*, and allows us to ascertain the detailed morphological structure of the female copulatory apparatus with spermathecae and the structure of the male hypopygium. The combination of detailed impression fossils with a specimen preserved three-dimensionally in resin has permitted study of the morphology of this Mesozoic fly genus almost to the level of modern genera. The paper includes descriptions of four new species of *Cretolimonia*: *C. lukashevichae* sp. nov., *C. pseudojurassica* sp. nov., *C. dayana* sp. nov. from sedimentary rocks, and *C. mikolajczyki* sp. nov. from Myanmar amber, supported with a key to all known species.

## Introduction

**Figure 1:**
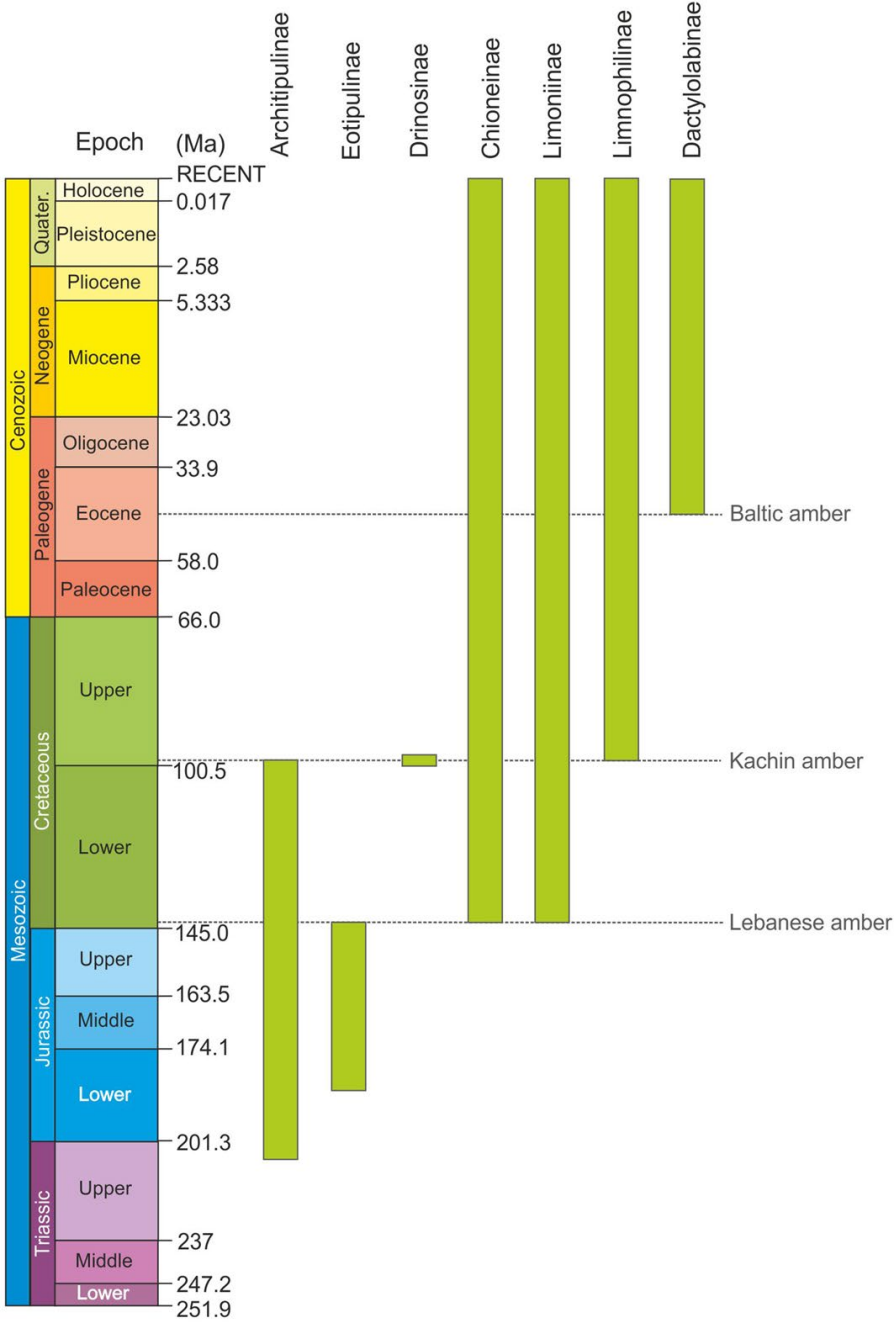
Geological ranges of subfamilies of the family Limoniidae.

The Limoniidae (limoniid craneflies), with over 10,000 described extant species, is the largest family in the dipteran infraorder Tipulomorpha, and one of the largest among all Nematocera. It is currently divided into seven subfamilies, three of which are extinct (Architipulinae, Eotipulinae, Drinosinae), and four extant: Limnophilinae, Chioneinae, Dactylolabinae, and Limoniinae (Fig. 1). Based on the fossil data known to date Architipulinae is considered the oldest group of Limoniidae^1–7^, with the oldest representative *Architipula youngi* Krzemiński, 1992 dating from the Late Triassic of North America^3^. This species was used to calibrate the age of the Tipulomorpha clade in the phylogenetic tree of Diptera^8^. During the Jurassic, the family Limoniidae, including Architipulinae, underwent rapid radiation expressed in an abundant and diverse assemblage of genera and species^3,7,9–16^. In the Cretaceous, Architipulinae gradually became extinct, while other limoniid families had appeared in that time.

The subfamily Architipulinae includes 11 fossil genera: *Architipula* Handlirsch, 1906^1^, *Protipula* Handlirsch, 1906^1^, *Mesotipula* Handlirsch, 1920^17^, *Paratipula* Bode, 1953^18^, *Haplotipula* Bode, 1953^18^, *Leptotipula* Bode, 1953^18^, *Ozotipula* Bode, 1953^18^, *Microtipula* Bode, 1953^18^, *Cretolimonia* Kalugina, 1986^2^, *Grimmenia* Krzemiński and Zessin, 1990^19^ and *Metarchilimonia* Blagoderov and Grimaldi, 2007^6^. Up until now, knowledge of the morphology of the subfamily has been based mainly on wing impressions in sedimentary rocks. No representatives of Architipulinae were known from amber and no male genitalia had been found. Similarly, the second fossil subfamily, Eotipulinae, is known mainly from wing impressions in Jurassic and Cretaceous sedimentary rocks^2,7^, although a female has been described from Lower Cretaceous Spanish amber^20^. In contrast, in the third fossil subfamily, Drinosinae, the morphology of both sexes has been elucidated through material preserved in mid-Cretaceous Myanmar amber^21^.

The Architipulinae material described here shows an exceptional level of preservation, allowing us to augment the diagnosis of the genus *Cretolimonia* and enriching our knowledge of the whole subfamily Architipulinae. The hypopygium (a modified abdominal segment associated with the genitalia and having a clasping function in the males) is described for the first time on the basis of a perfectly preserved imprint in the rock, as well as the first representative of the subfamily from a fossil resin. Four new species of *Cretolimonia* are described, and a key to all known species is provided.

## Results

### Systematic palaeontology

- Order Diptera Linnaeus, 1758
- Infraorder Tipulomorpha Rodendorf, 1961^22^
- Family Limoniidae Speiser, 1909^23^
- Subfamily Architipulinae Handlirsch, 1906^1^
-                Genus *Cretolimonia* Kalugina, 1986^2^

#### Type species

*Cretolimonia popovi* Kalugina, 1986^2^: figs. 87a, b: Gurvan-Ereny-Nuru (West Mongolia), Early Cretaceous. Genus description based on a wing fragment.

#### Species included

Table 1.

**Table 1:** List of species of *Cretolimonia* known from the fossil record.

Species	Time scale	Type of material	Locality
*Cretolimonia popovi* Kalugina, 1986^2^	Early Cretaceous	Imprint	West Mongolia
*Cretolimonia jurassica* Lukashevich, 2009^7^	Late Jurassic	Imprint	Mongolia, Shar Teg
*Cretolimonia pygmea* Lukashevich, 2009^7^	Late Jurassic	Imprint	Mongolia, Shar Teg
*Cretolimonia excelsa* Gao et al., 2015^24^	Middle Jurassic	Imprint	Inner Mongolia, Daohugou
*Cretolimonia lukashevichae* sp. nov.	Early Cretaceous	Imprint	Transbaikalia, (Shevia)
*Cretolimonia pseudojurassica* sp. nov.	Jurassic/Cretaceous boundary	Imprint	Transbaikalia, (Shevia)
*Cretolimonia dayana* sp. nov.	Jurassic/Cretaceous boundary	Imprint	Transbaikalia, (Daya)
*Cretolimonia mikolajczyki* sp. nov.	mid-Cretaceous	Amber	Northern Myanmar

#### Amended diagnosis

The genus is distinguished from all Limoniidae by the characteristic wing venation pattern: vein Sc ends near Rs bifurcation, four radial veins present (R_1_, R_3_, R_4_ and R_5_), cross-vein r-r (R_2_) atrophied, and vein R_3_ very short, slightly curved; basal medial vein (Mb) well visible, long, all four medial veins present, d-cell closed, cross-vein m-cu located at the distal part of the d-cell base or sometimes in the middle (*C. excelsa*); ovipositor short, strongly curved dorsally; three small spermathecae present; male hypopygium with outer gonostylus strongly hooked; inner gonostylus lobed; aedeagus narrow and slightly curved, parameres large, triangular, very dilated at base.

#### Remarks

**Figure 2:**
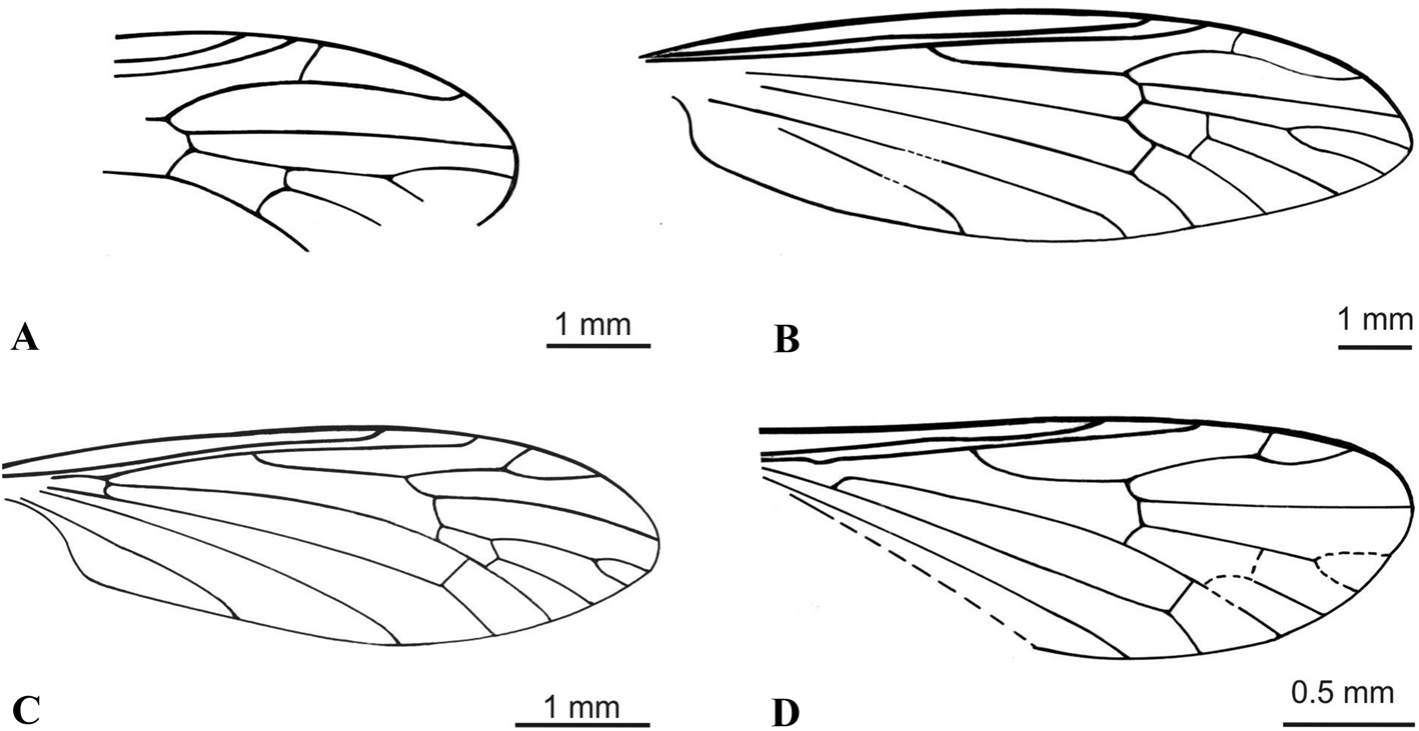
Wing venation of previously described species from genus *Cretolimonia* redrawn from original papers: (**A**) *Cretolimonia popovi* Kalugina, 1986; (**B**) *Cretolimonia excelsa* Gao et al., 2015; (**C**) *Cretolimonia jurassica* Lukashevich, 2009; (**D**) *Cretolimonia pygmaea* Lukashevich, 2009.

The genus *Cretolimonia* was included in the subfamily Architipulinae based on complex features which define this subfamily, i.e.: four radial and four medial veins reaching the wing margin, closed d-cell; cross vein m-cu located at the end of d-cell. The absence of cross-vein r-r (R_2_) distinguishes the genus *Cretolimonia* among other genera of the subfamily Architipulinae. So far, four species from the Middle Jurassic to mid-Cretaceous have been included in the genus (Table 1). For the purpose of the key, their wings are shown in Fig. 2A-D.

**Key to species in genus** ***Cretolimonia*** **:**

**Figure 3:**
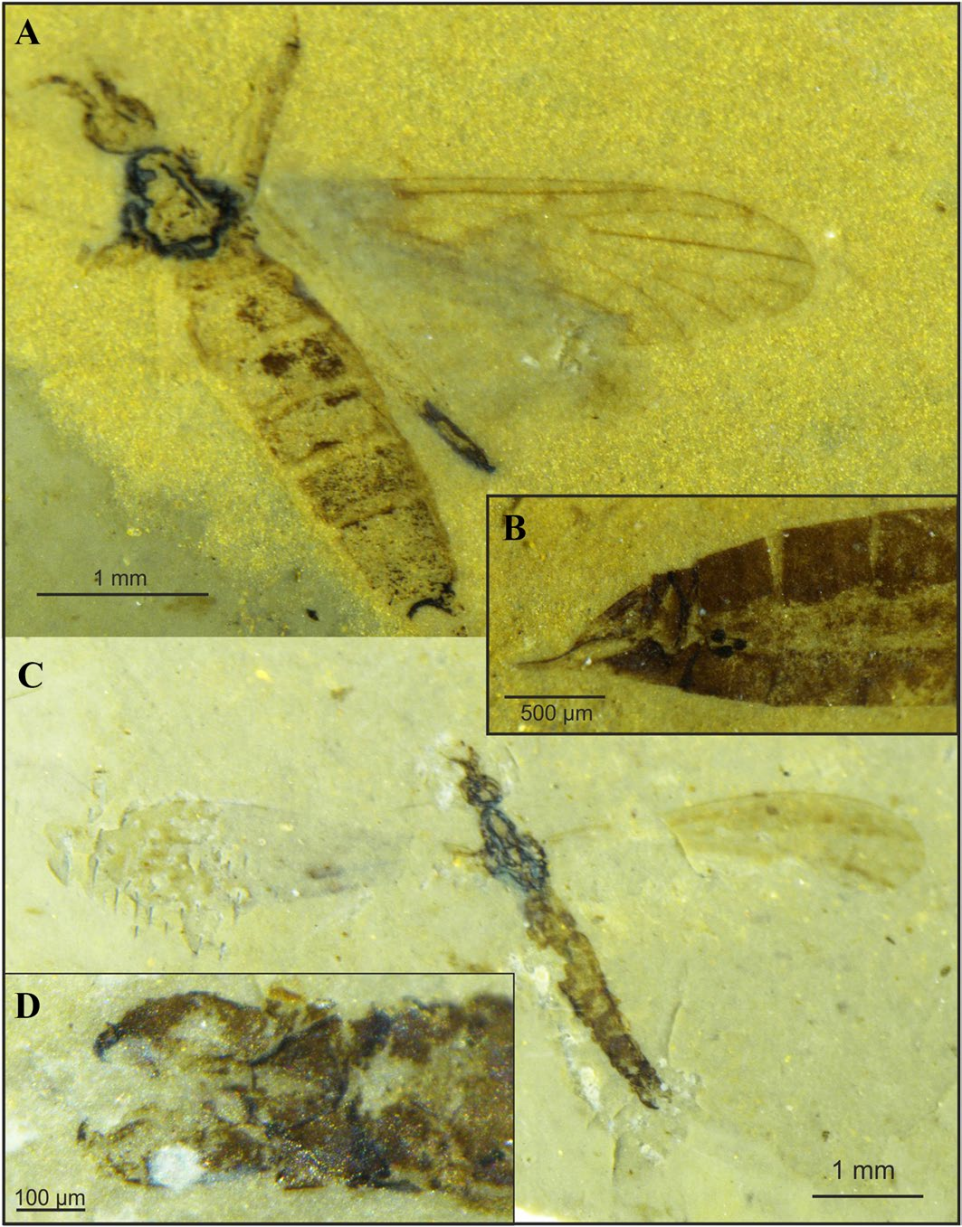
*Cretolimonia dayana* sp. nov.: (**A**) habitus of female, holotype No. 3063/1208; (**B**) female abdomen, No. 3063/399; (**C**) habitus of male, No. 3063/1079; (**D**) male hypopygium, No. 3063/1079.

**Figure 4:**
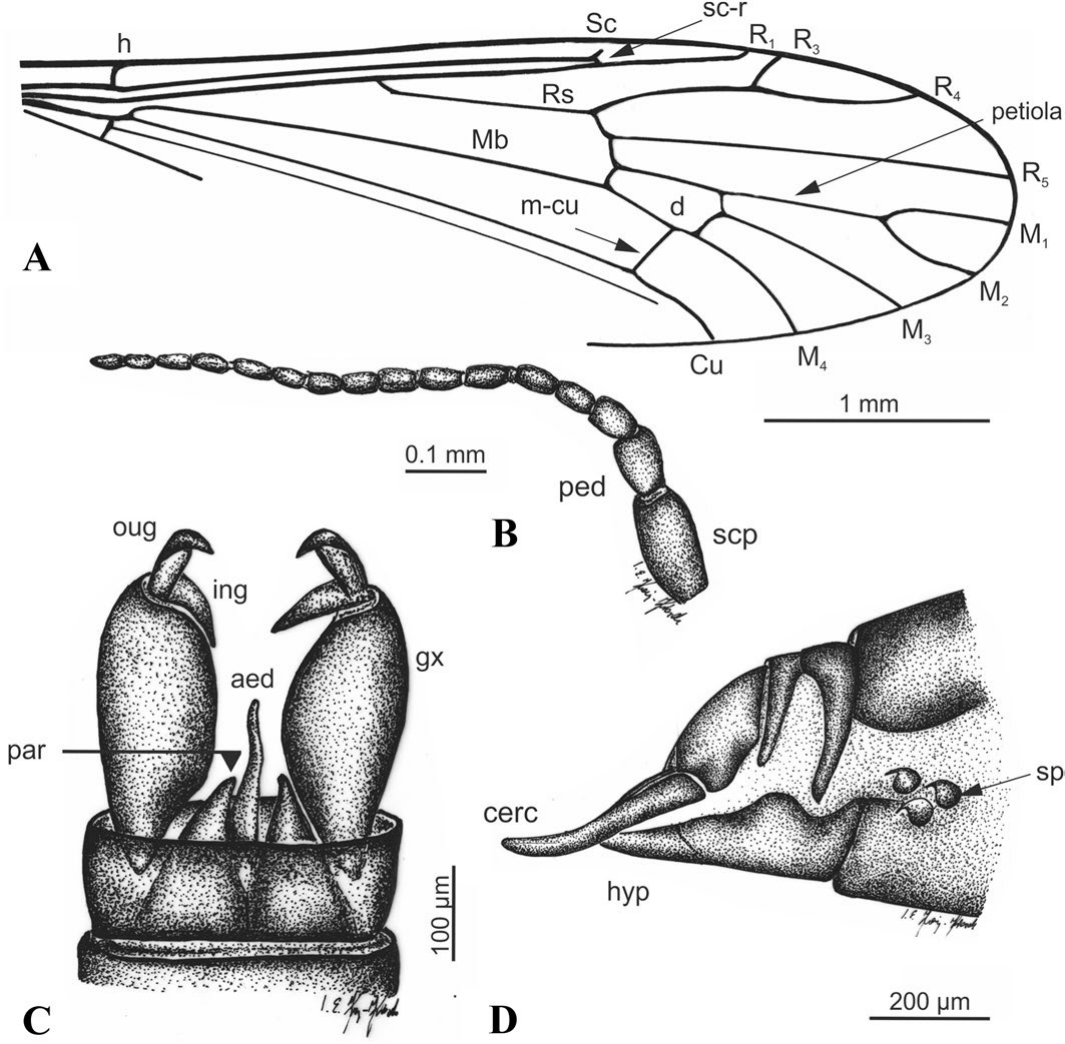
*Cretolimonia dayana* sp. nov.: (**A**) wing venation, holotype No. 3063/1208; (**B**) antenna, holotype No. 3063/1208; (**C**) male hypopygium (reconstruction), No. 3063/1079; (**D**) female genitalia, No. 3063/399. Abbreviation: *aed* aedeagus, *cer* cercus, *gx* gonocoxite, *hyp* hypogenium, *ing* inner gonostylus, *oug* outer gonostylus, *par* parameres, *ped* pedicel, *scp* scapus, *sp* spermathecae.

- 1. wing narrow, at least 3x longer than width .......... 2
- wing wide, about 2.5x longer than width .......... 7
- 2. Sc ends far beyond the Rs fork .......... 3
- Sc ends before or opposite the Rs fork .......... 4
- 3. M_1_ twice as long as upper edge of d-cell (Fig. 2A) .......... ***C. popovi*** Kalugina, 1986^2^
- M_1_ only slightly longer than upper edge of d-cell (Fig. 2B) .......... ***C. excelsa*** Gao et al., 2015^23^
- 4. Sc ends opposite Rs fork .......... ***C. dayana* sp. nov.**
- Sc ends in front of the Rs fork .......... 5
- 5. d-cell very small, no more than 1/10 of the wing length (Fig. 2C) .......... ***C. jurassica*** Lukashevich, 2009^7^
- d-cell large, no more than 1/7 of the wing length .......... 6
- 6. R_4_ almost equal to the length of R_2+3+4_ .......... ***C. pseudojurassica* sp. nov.**
- R_2+3+4_ about 1/3 longer than R_4_ (Fig. 2D) .......... ***C. pygmea*** Lukashevich, 2009^7^
- 7. petiola longer than M_1_; m-cu almost 2/3 along lower part of d-cell .......... ***C. lukashevichae* sp. nov.**
- petiola only about 1/3 as long as M_1_; m-cu just before the M_3+4_ bifurcation .......... ***C. mikolajczyki* sp. nov.**
- *Cretolimonia dayana* Kopeć n. sp.
- (Fig. 3-4)

#### Etymology

The name was established from the site where this species was discovered.

#### Material examined

Holotype No. 3063/1208, female (Fig. 3A,B). Additional material: 3063/399, female; 3063/1072, female; 3063/1079, male (Fig. 3C,D). Specimens come from Daya (Transbaikalia, Russia), Jurassic/Cretaceous boundary, housed in the Borissiak Palaeontological Institute, Russian Academy of Sciences, Moscow, Russia (PIN).

#### Diagnosis

Wing narrow, about 3.3 times longer than its width; venation differs in the proportions of the individual veins from all species in this genus. Sc ends opposite the fork of Rs, d-cell long, narrow, expanded at distal part, no more than 1/9 of the wing length; m-cell lies at 2/3 length of the base of the d-cell; gonocoxites short, broad; outer gonostylus short, strongly chitinized, significantly hooked at end; inner gonostylus delicate, lobed; aedeagus long, narrow, slightly curved; parameres shorter than penis, broad at base.

#### Description

Wing length ca. 4.6 mm, width 1.4 mm (Fig. 4A); *Head*. Antennae with 16 segments, scapus tubular, half as long as its width, pedicel barrel-shaped, only slightly wider than scapus; flagellum with 14 flagellomeres, flagellomeres ovoid, gradually shortening, basal segment of flagellum expanded in lower part, last segment small and round, on all flagellomeres there are bristles shorter than width of segment on which they are located, on last segment 3 or 4 short bristles; clypeus 4-segmented, short, and the last segment almost equal in length to the penultimate one (Fig. 4B). *Thorax*. Wing narrow, about 3.3 times as long as its width; vein Sc ends opposite the Rs fork; cross-vein sc-r is about twice its length before the end of Sc; R_1_ ends opposite the R_2+3+4_ bifurcation at R_3_ and R_4_, and R_2_ (r-r) completely disappears; Rs about 1/4 longer than R_2+3+4_; R_3_ short, strongly sickle-shaped, slightly inclined, less than 1/3 length of R_2+3+4_, R_4_ about 1/4 shorter than Rs and equal in length to R_2+3+4_; four medial veins present, petiola ca. 1/4 longer than M_1_ and 1/3 longer than upper edge of d-cell; d-cell long, ca. 1/9 of wing length; cross-vein m-cu lies at 2/3 length of d-cell base; A_2_ vein not visible; legs long and delicate, tibial spurs absent. *Abdomen*. *Male*. Gonocoxites short, broad; outer gonostylus short, strongly chitinized, strongly hooked at the end; inner gonostylus delicate, lobed; penis long, narrow, slightly sigmoidally curved; parameres broad at base, shorter than aedeagus (3D,4C). *Female*. Ovipositor short, slightly curved dorsally; three small spermathecae (Fig. 3B, 4D).

#### Remarks

The male hypopygium is very well preserved in specimen No. 3063/1079 (Fig. 3D), being only slightly deformed by elongation of the left gonocoxite during fossilization. In previously described species of this genus the copulatory apparatus has not been preserved. The female ovipositor (Figs. 3B, 4D) is preserved almost perfectly in specimen No. 3063/399 with three small spermathecae present and well visible. The spermathecae are identical to those of most species in the subfamily Limnophilinae.

**Figure 5:**
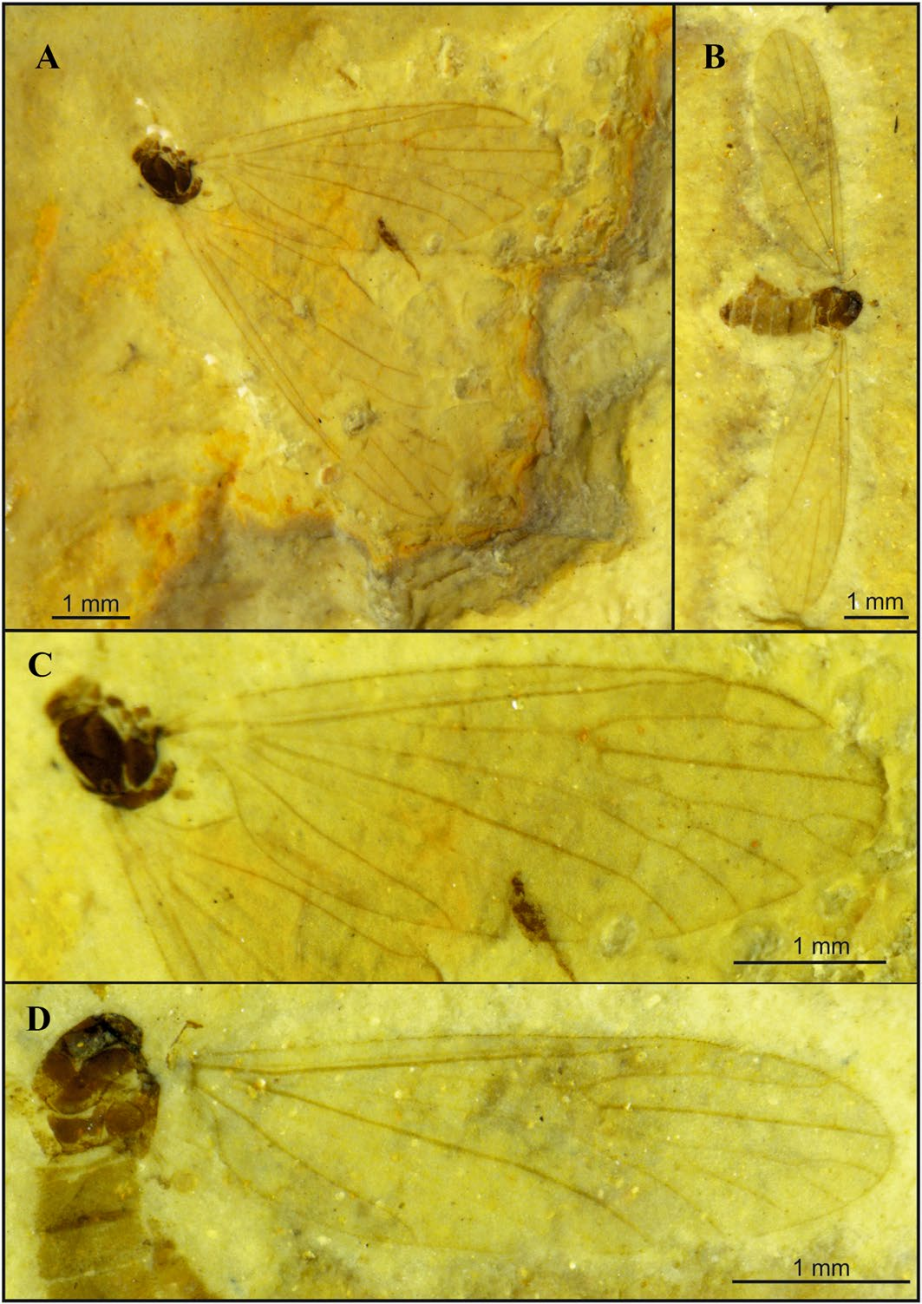
Holotypes of new *Cretolimonia* species: (**A**, **C**) *Cretolimonia lukashevichae* sp. nov. No. 3795/637, (**A**) habitus of holotype, (**C**) wing; (**B**, **D**) *Cretolimonia pseudojurassica* sp. nov. No. 3795/628, (**B**) habitus of holotype, (**D**) wing.

-                *Cretolimonia lukashevichae* Kopeć and Krzemiński n. sp.
-                                 (Fig. 5A,C 6A)

**Figure 6:**
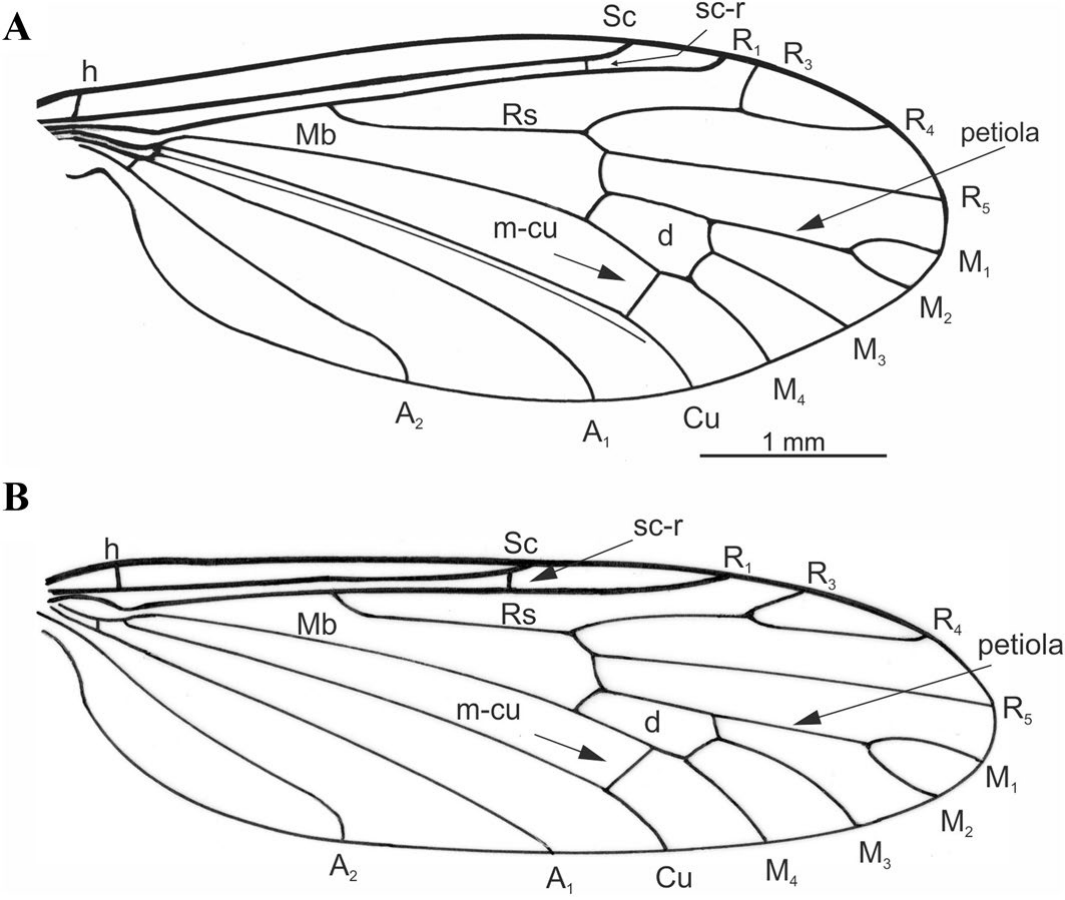
Holotypes of new *Cretolimonia* species: (**A**) *Cretolimonia lukashevichae* sp. nov., No. 3795/637; (**B**) *Cretolimonia pseudojurassica* sp. nov., No. 3795/623—wing venation.

#### Etymology

The species name is dedicated to Dr. Elena D. Lukashevich, who is involved in the study of fossil Diptera.

#### Material examined

Holotype No. 3795/637 sex unknown (Fig. 5A); paratype No. 3795/611 (sex unknown, hind wing preserved), Shevia (Transbaikalia, Russia), Jurassic/Cretaceous boundary; housed in the Borissiak Palaeontological Institute, Russian Academy of Sciences, Moscow, Russia (PIN).

#### Diagnosis

Wing broad, 2.5 times as long as its width; Sc ends distinctly beyond fork of Rs; petiola more than half as long as M_1_ and 1/4 as long as upper margin of d-cell; m-cu lies nearly at 2/3 length of lower part of d-cell.

#### Description

Wing length 5.0 mm (right wing), width 2.0 mm, wing 2.5 times longer than its width (Fig. 5C, 6A); Sc ends distinctly beyond the Rs fork; cross-vein sc-r twice its length before the end of Sc; R_1_ ends before the R_2+3+4_ bifurcation at R_3_ and R_4_; R_2_ completely atrophied; Rs ca. 1/4 longer than vein R_2+3+4_; R_3_ short, almost vertical, constitutes ca. 1/3 length of R_2+3+4_; R_4_ ca. 1/4 shorter than R_2+3+4_; four medial veins present, petiola half longer than M1 and 1/4 longer than upper edge of d-cell; d-cell trapezoidal, relatively large, constituting 1/7 of wing length, cross-vein m-cu lies at 2/3 length of base of d-cell, far before bifurcation of M_3+4_ into M_3_ and M_4_; A_2_ vein almost straight.

#### Remarks

The holotype specimen shows two well-preserved wings and a fragment of the thorax. The left wing is longer than the right one, most probably deformed (elongated) during the fossilization process. Such a phenomenon has been observed in different groups of insects^25,26^. The right wing retained its normal structure.

-                *Cretolimonia pseudojurassica* Krzemiński sp. nov.
-                                 (Fig. 5B,D 6B)

#### Etymology

The species name emphasizes the similarity to another species in the genus, *Cretolimonia jurassica* Lukashevich, 2009^7^.

#### Material examined

Holotype No. 3795/628 (Fig. 5B), sex unknown, only a single wing preserved. Additional material: 3795/633; 3795/638. Shevia (Transbaikalia, Russia), Jurassic/Cretaceous boundary. Housed in the Borissiak Palaeontological Institute, Russian Academy of Sciences, Moscow, Russia (PIN).

#### Diagnosis

Wing narrow, nearly 3.3 times as long as its width; vein Sc ends distinctly before fork of Rs; R_4_ equal in length to R_2+3+4_; M_1_ slightly shorter than petiola and equal in length to upper margin of d-cell.

#### Description

Wing well-preserved with clearly visible veins, 5.1 mm long and 1.6 mm wide, almost 3.3 times as long as its width (Figs. 5D, 6B); Sc ends distinctly before bifurcation of Rs; cross-vein sc-r about its length before end of Sc; R_1_ ends before bifurcation of R_2+3+4_ into R_3_ and R_4_; R_2_ fully atrophied; Rs about 1/3 longer than R_2+3+4_; R_3_ short, almost parallel to R_4_, ca. 1/3 length of R_2+3+4_; R4 equal in length to R_2+3+4_; four medial veins present, petiola half longer than M1 and equal in length to upper edge of d-cell, d-cell trapezoidal, relatively large, constitutes ca. 1/6 length of wing; m-cu lies at 2/3 length of base of d-cell, far before bifurcation of M_3+4_ into M_3_ and M_4_; vein A_2_ slightly wavy.

#### Remarks

The wing venation resembles that of *C. jurassica* but significantly differs from that species in the structure of the d-cell and the proportions of the medial veins.

**Figure 7:**
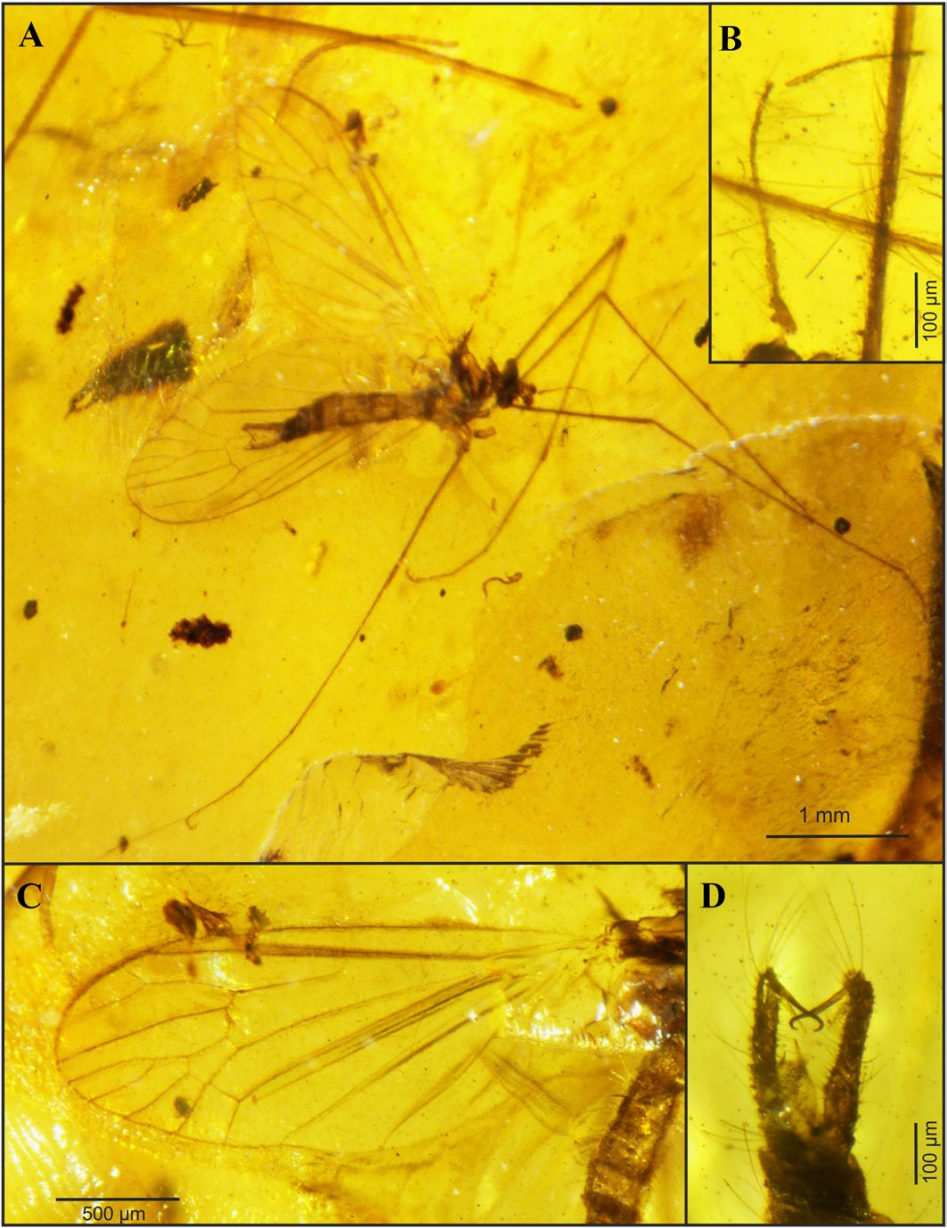
*Cretolimonia mikolajczyki* sp. nov., holotype No. MP/4082: (**A**) habitus of male; (**B**) antennae; (**C**) wing; (**D**) male hypopygium.

**Figure 8:**
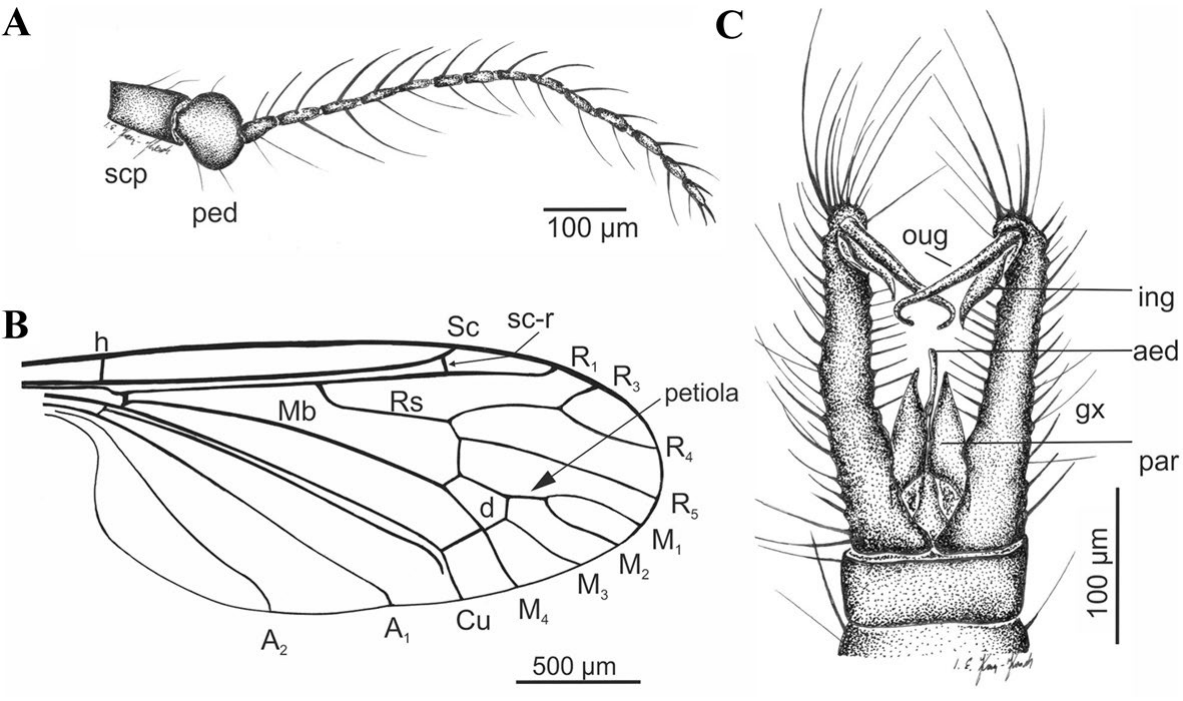
*Cretolimonia mikolajczyki* sp. nov. Holotype No. MP/4082: (**A**) antenna; (**B**) wing venation; (**C**) male hypopygium. Abbreviation: *aed* aedeagus, *gx* gonocoxite, *ing* inner gonostylus, *oug* outer gonostylus, *par* parameres, *ped* pedicel, *scp* scapus.

-                *Cretolimonia mikolajczyki* Kopeć, Krzemiński, Soszyńska-Maj sp. nov.
-                                 (Figs. 7-8)

#### Etymology

We dedicate the species to the memory of a great and regrettably late colleague, the well-known Polish dipterologist Dr Waldemar Mikołajczyk.

#### Material examined

Holotype No. MP/4082, male (Fig. 7A); Kachin amber (Northern Myanmar); mid-Cretaceous, earliest Cenomanian. Housed in the Institute of Systematics and Evolution of Animals, Polish Academy of Sciences (ISEA PAS).

#### Diagnosis

Wing broad, about 2.3 times as long as its width; wing venation differs in proportions of individual veins from all species in this genus; vein Sc ends opposite the fork of Rs, d-cell trapezoidal and distinctly expanded at the tip, constituting 1/10 of the wing length; petiola constitutes only about 1/3 of M1; cross-vein m-cu just before the fork M3+4 on M3 and M4; gonocoxites long, narrow, with numerous bristles, outer gonostylus long, narrow, strongly hooked at the tip; inner gonostylus delicate, lobed, elongated at the tip.

#### Description

Well-preserved specimen. Wing length 2.6 mm; width 1.2 mm; body length 2.8 mm. *Head*. Considerably wider than its length; antennae 16 segmented, scapus large, tubular half as long as its width, pedicel round, almost half as wide as scapus; flagellum 14 segmented, the first basal segment expanded in the lower part, the last small and rounded; bristles are present on all the flagellomeres, on the three basal segments bristles 2.5-3 times as long as the width of the segment, on the other flagellomeres bristles are shorter, 1.5-2 times as long as the width of the segment, on the last segment 3 or 4 short bristles (Fig. 7B, 8A). Palpi invisible. *Thorax*. Wing about 2.3 times as long as its width (Fig. 7C, 8B), additionally expanded in the anal field, vein Sc ends opposite bifurcation of Rs; cross-vein sc-r is about its length opposite the end of Sc; R_1_ ends opposite bifurcation of R_2+3+4_ into R_3_ and R_4_, R_2_ (r-r) fully atrophied; Rs about 1/4 longer than R_2+3+4_; R3 short, slanting, less than 1/3 length of R_2+3+4_; R4 only slightly shorter than R_2+3+4_; four medial veins present, and vein M_1_ 2.5 times longer than petiola and twice as long as upper edge of d-cell; d-cell trapezoidal, small, constituting only 1/10 of wing length; cross-vein m-cu at end of d-cell, just before M_3+4_ bifurcates into M_3_ and M_4_; A_2_ vein strongly waved. Legs long, delicate, tibial spurs absent. *Abdomen*. Hypopygium (Fig. 7D, 8C): gonocoxites long, narrow, with numerous bristles, at end bearing a bunch of long bristles; outer gonostylus strongly chitinized, long, narrow, strongly hooked at end; inner gonostylus delicate, lobed, elongate at the end; penis long, narrow, slightly curved; parameres shorter than penis, broad at base, similar to parameres in *Cretolimonia dayana *sp. nov..

#### Remarks

*Cretolimonia mikolajczyki* sp. nov. is the first representative of the genus, and of the Architipulinae, found in fossil resin. Its excellent preservation permits detailed examination of morphological characters, especially the structure of the antennae and copulatory apparatus.

## Discussion

Representatives of the infraorder Tipulomorpha were already present among the oldest fossil Diptera specimens from the early Middle Triassic (Anisian), ca. 245 Ma, from Arzviller (Vosges Mts., France)^3,5,15,27,28^. *Archilimonia vogesiana* Krzemiński and Krzemińska, 2003^5^ and *A. krzeminski* Lukashevich and Ribeiro, 2019^16^ belong to the fossil family Archilimoniidae. Archilimoniidae was included as a subfamily within the family Limoniidae, which we consider erroneous since the wing venation more closely resembles that of Pediciidae, especially in the radial field^16^. This being the case, the oldest known representative of the Limoniidae would be *Architipula youngi* Krzemiński, 1992^3^ from the Late Triassic of North America (ca. 220 Ma)^6^ used for age calibration of Tipulomorpha^8^. It should be noted that the first 100 million years of dipteran, and hence tipulomorph, evolution is documented only by impression fossils, mainly wings^28^. The earliest examples of Limoniidae revealing three-dimensional structure have been recovered from Lower Cretaceous Lebanese amber, but such inclusions are very few in number^29–31^.

The specimens described here are of paramount importance for understanding the morphology of Architipulinae (Fig. 7A), and thus shed new light on the early evolution of flies of the suborder Tipulomorpha. The male genitalia of these flies are usually so severely deformed during fossilization that their precise structure and spatial arrangement cannot be determined. Although the preservation of *Cretolimonia dayana* sp. nov. is very good, being an impression fossil, it is only possible to obtain information in two dimensions. Therefore, finding a male of the same genus (*C. mikolajczyki* sp. nov.) in mid-Cretaceous amber presented a unique opportunity to fully reconstruct the genital anatomy, and thus to verify the structure of the hypopygium of *C. dayana *sp. nov. (Fig. 3B,4D). In sedimentary rocks, the genitalia of females are much better preserved, as they are usually strongly chitinized; in favourable circumstances even spermathecae are visible, and sometimes also genital plates^26^. However, so far, the number of spermathecae has not been known for the oldest known Limoniidae, the subfamilies Architipulinae or Eotipulinae. The well-preserved material from the Daya site allowed us to reconstruct the genitalia of the female *C. dayana* sp. nov. and to determine the number of spermathecae. The spermathecae are identical to those of most species in the family Limoniidae.

Most genera of Limoniidae have a full set of five radial veins, including the R_2_ vein, which takes the form of a cross-vein r-r. However, as far back as the Early Jurassic, a number of genera had appeared in which the cross-vein r-r (R_2_) had disappeared (i.e., atrophied) and one of these is the genus *Cretolimonia*. The disappearance of this cross-vein is observed also in some other Limoniidae, for example, in the subfamily Chioneinae, in the modern genera *Gonomyia* Meigen, 1818 and *Rhabdomastix* Skuse, 1890. There are, however, significant differences in other sectors of the wing in these genera; only three medial veins are always present, and the cross-vein m-cu is located in the anterior half of the d-cell, usually near the bifurcation of M_3+4_ into M_3_ and M_4_.

New material described here enabled us to characterize the morphology of the oldest (at least in the geological sense), extinct group of the Limoniidae; these features will be of great importance when introduced to the phylogenetical analysis of this large dipteran family.

## Materials and methods

### Geological context

**Figure 9:**
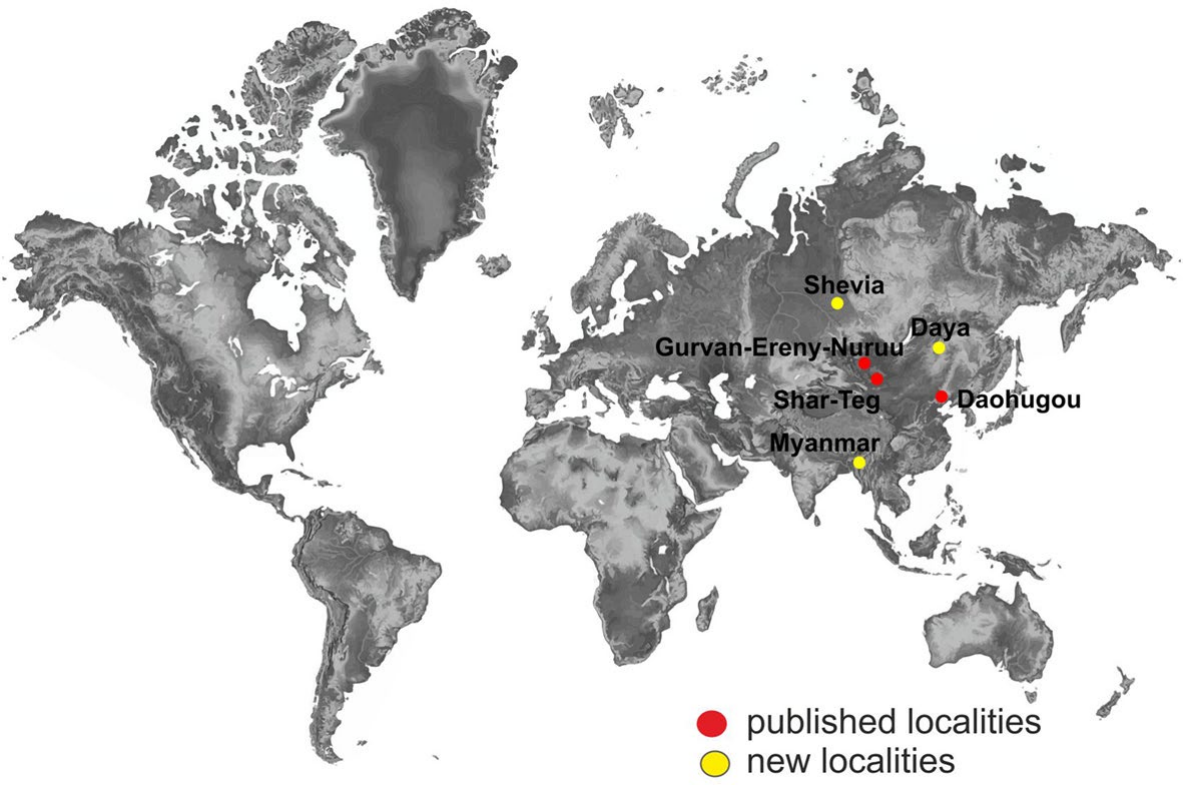
Map of localities yielding fossils of the genus *Cretolimonia,* yellow type—new localities.

This study was based on impression fossils and an amber inclusion (Fig. 9). The rock material comes from two sites, Shevia and Daya, in the Shelopuginsky District of the Chita region of Transbaikalia (Russia). Shevia (Dain Formation), dated Early Cretaceous, is located on the right bank of the Shevia River, 3 km below Shevia village, 2 km above the confluence of the Shevia and Shiviinsky Bumulei Rivers. The Daya site is located on the left bank of the Daya River above the Shevia Valley. It exposes sediments of the Glushkovo Formation, which are imprecisely dated but likely to lie close to the Jurassic/Cretaceous boundary^32–34^.

The investigated amber inclusion derives from a former amber mine located near Danai (Tanai) Town (approximately at 26° 150″ N, 96° 340″ E) in the Hukawng Valley, state of Kachin in northern Myanmar. Radiometric U-Pb zircon dating of the volcaniclastic matrix of the amber produced an age of 98.79 ± 0.62 million years (earliest Cenomanian)^29,35^.

### Specimen repository

All the specimens studied in the course of this work are deposited permanently in publicly owned collections in national museums. The Myanmar amber inclusion, the holotype of *C. mikolajczyki* sp. nov., No. MP/4082, is housed at the collection of the Institute of Systematics and Evolution of Animals, Polish Academy of Sciences (ISEA PAS). It was acquired in 2016, before the armed conflict and the escalation of the ethnic strife in the area, in full compliance with the International Code of Zoological Nomenclature and Statement of the International Palaeoentomological Society^36^. The holotype of *Cretolimonia dayana* sp. nov. No. 3063/1208 and additional material 3063/399, 3063/1072; 3063/1079, holotype of *C. lukashevichae* sp. nov. No. 3795/637, paratype No. 3795/611 and holotypes of *C. pseudojurassica* sp. nov. No. 3795/623, along with additional material 3795/633; 3795/638 are housed in the collection of Borissiak Palaeontological Institute, Russian Academy of Sciences, Moscow, Russia (PIN).

## Methods

Specimens were studied using a Nikon SMZ25 stereomicroscope under reflected light, and photographs taken with a Nikon DSRi2 digital camera. The surfaces of rock specimens were wetted with 98% ethyl alcohol to improve the image contrast. Drawings were executed on the basis of the photographs with permanent referral to the specimens. The terminology of wing venation follows^11,27^. Geological ranges of subfamilies of the family Limoniidae are based on published data^1,3,29–31,37^.
